# Pooled Plasmid Sequencing Reveals the Relationship Between Mobile Genetic Elements and Antimicrobial Resistance Genes in Clinically Isolated *Klebsiella pneumoniae*

**DOI:** 10.1016/j.gpb.2020.12.002

**Published:** 2020-12-30

**Authors:** Yan Jiang, Yanfei Wang, Xiaoting Hua, Yue Qu, Anton Y. Peleg, Yunsong Yu

**Affiliations:** 1 Department of Infectious Diseases, Sir Run Run Shaw Hospital, Zhejiang University School of Medicine, Hangzhou 310016, China; 2 Key Laboratory of Microbial Technology and Bioinformatics of Zhejiang Province, Hangzhou 310016, China; 3 Biomedicine Discovery Institute, Department of Microbiology, Faculty of Medicine, Nursing and Health Sciences, Monash University, Melbourne 3800, Australia; 4 Department of Infectious Diseases, The Alfred Hospital and Central Clinical School, Monash University, Melbourne 3004, Australia

**Keywords:** Horizontal transfer profile, Transconjugant, Single-molecule real time sequencing, Insertion sequence, Plasmid

## Abstract

Plasmids remain important microbial components mediating the horizontal gene transfer (HGT) and dissemination of antimicrobial resistance. To systematically explore the relationship between mobile genetic elements (MGEs) and antimicrobial resistance genes (ARGs), a novel strategy using single-molecule real-time (SMRT) sequencing was developed. This approach was applied to pooled conjugative plasmids from clinically isolated multidrug-resistant (MDR) *Klebsiella pneumoniae* from a tertiary referral hospital over a 9-month period. The conjugative plasmid pool was obtained from transconjugants that acquired antimicrobial resistance after plasmid conjugation with 53 clinical isolates. The plasmid pool was then subjected to SMRT sequencing, and 82 assembled plasmid fragments were obtained. In total, 124 ARGs (responsible for resistance to β-lactam, fluoroquinolone, and aminoglycoside, among others) and 317 MGEs [including transposons (Tns), insertion sequences (ISs), and integrons] were derived from these fragments. Most of these ARGs were linked to MGEs, allowing for the establishment of a relationship network between MGEs and/or ARGs that can be used to describe the dissemination of resistance by mobile elements. Key elements involved in resistance transposition were identified, including IS*26*, Tn*3*, IS*903B*, IS*Ecp1*, and IS*Kpn19*. As the most predominant IS in the network, a typical IS*26*-mediated multicopy composite transposition event was illustrated by tracing its flanking 8-bp target site duplications (TSDs). The landscape of the pooled plasmid sequences highlights the diversity and complexity of the relationship between MGEs and ARGs, underpinning the clinical value of dominant HGT profiles.

## Introduction

*Klebsiella pneumoniae* is a major opportunistic pathogen causing hospital-acquired infections including pneumonia, urinary tract infections, septicaemia, and soft-tissue infections [1], [2], [3]. Broad-spectrum antimicrobials have been intensively used for the treatment of hospital-acquired infections and linked to the emergence and domination of *K*. *pneumoniae* multidrug resistance (MDR, resistance to ≥ 3 antimicrobial classes) in many hospital wards [4]. Indeed, acquisition of novel antimicrobial resistance genes (ARGs) frequently occurs in clinically isolated *K*. *pneumoniae* [4], [5].

The dissemination of antimicrobial resistance is often mediated by horizontal gene transfer (HGT), in which conjugative plasmids play an important role [6], [7], [8]. These plasmids are often large in size (> 25 kb) and able to horizontally transmit genes through conjugation in nosocomial environments. A conjugative plasmid can act as a “vehicle”, which carries resistance genes and other functional modules and transfers resistance determinants with mobile genetic elements (MGEs), including integrons, transposons (Tns), and insertion sequences (ISs). Therefore, conjugative plasmids are now central to the rapid global emergence of antimicrobial resistance [9], [10], [11].

ISs are the simplest mobile elements present in bacterial genomes and are also frequently found in plasmids derived from members of Enterobacteriaceae. ISs are typically short sequences comprising terminal inverted repeats (TIRs) at the ends and an open reading frame (ORF) which encodes the transposase essential for mobility [12]. Donor ISs normally attack the target site to generate a short nucleotide sequence of direct repeats (target site duplication, TSD) to achieve its transposition. Most ISs identified to date belong to RNase H or DDE transposase superfamily. Some ISs undergo transposition using a cut-and-paste mechanism, whereas others use a copy-in or so-called replicative transposition mechanism, whereby a second copy of the IS is utilized at the target site and the original copy remains intact [12], [13].

**Figure 1:**
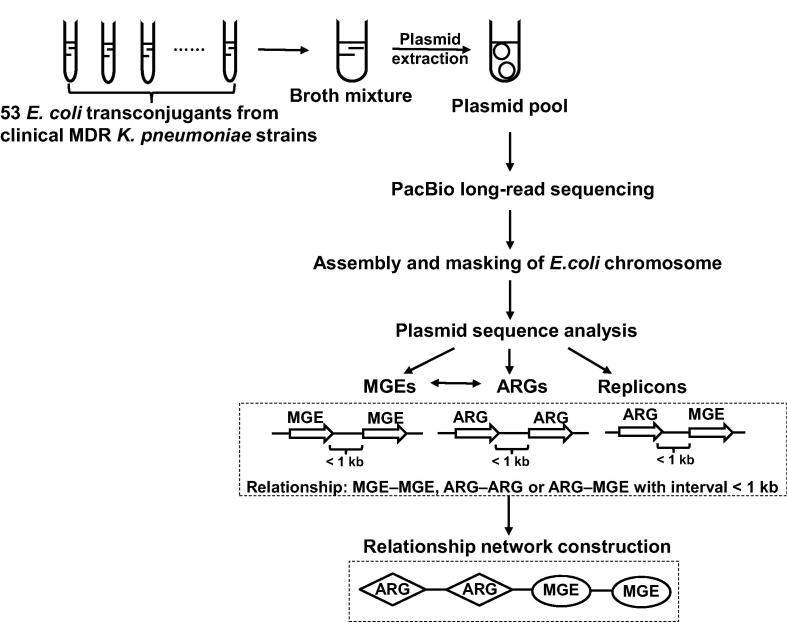
**Overall strategy and workflow of our study** The strategy developed in our study is shown, from extracting the pooled plasmids of *E*. *coli* transconjugants to plasmid sequence analysis including ARG and MGE relationship network construction. MDR, multidrug resistance; MGE, mobile genetic element; ARG, antimicrobial resistance gene.

In this study, we carried out a single-molecule real-time (SMRT) sequencing assay for pooled conjugative plasmids representing the landscape of plasmids from a collection of 53 MDR *K*. *pneumoniae* clinical isolates. The genetic diversity of clinical MDR plasmids was analyzed using data from large-scale sequencing. Horizontally transferred elements in plasmids were surveyed to facilitate the discovery of novel pathways in transfer of antimicrobial resistance determinants. The workflow of our study is shown in Figure 1.

## Results

### The diversity of clinical ***K*****.*****pneumoniae*** isolates and plasmid size

Pulsed-field gel electrophoresis (PFGE) typing of 53 clinical *K*. *pneumoniae* isolates confirmed the diversity of the bacterial collection used in this study (data not shown). Most isolates (45/53) were allocated to different PFGE clades, with eight isolates constituting two clades (one with 3 strains and the other with 5 strains). Notably, isolates of the same clade possessed conjugative plasmids of different sizes, as revealed by the S1 nuclease digestion assay, and the average plasmid size in all isolates was approximately 135 kb (in the range of 34 kb to 355 kb) (Figure S1).

### Antimicrobial susceptibility profiles of clinical **K**. **pneumoniae** isolates

All 53 *K*. *pneumoniae* isolates showed MDR against antimicrobial agents of different classes, such as β-lactams, aminoglycosides, and fluoroquinolones (Figure S2). Most of these clinical isolates (> 90%) were resistant to ampicillin, piperacillin, cefazolin, cefuroxime, and ampicillin/sulbactam, and able to transfer their resistance to transconjugants via ampicillin-selected conjugation.

### Replicons, ARGs, and MGEs in pooled plasmid sequencing data

The pool of 53 conjugative plasmids was sequenced using the SMRT technique, and 50,719 raw reads were received, with a mean read length of 13,671 bp. The raw reads were assembled into 394 unitigs, with maximum and N50 unitig lengths of 331,152 bp and 22,800 bp, respectively. After removing recipient chromosome sequences, 82 unitigs were retained as plasmid sequence fragments and used for further analysis. Among the 82 unitigs, eight were successfully cyclized into complete plasmids. The maximum plasmid unitig length was 281,339 bp and the N50 plasmid unitig length was 48,395 bp (Table 1).

**Table 1:** SMRT sequencing raw data and assembly metrics

*Note*: N50 defines a weighted median statistic, *i.e.*, the shortest sequence length at 50% of the entire assembly. SMRT, single-molecule real-time; ORF, open reading frame; ARG, antimicrobial resistance gene; MGE, mobile genetic element; Tn, transposon; IS, insertion sequence.

Sixteen replicons were identified to belong to ten different classes, including IncL/M, IncFIB(K), IncFII, IncFII(K), IncA/C2, IncR, IncN, IncI1, IncI2, and IncHI1B, with the IncFII(K) class most frequently identified.

**Figure 2:**
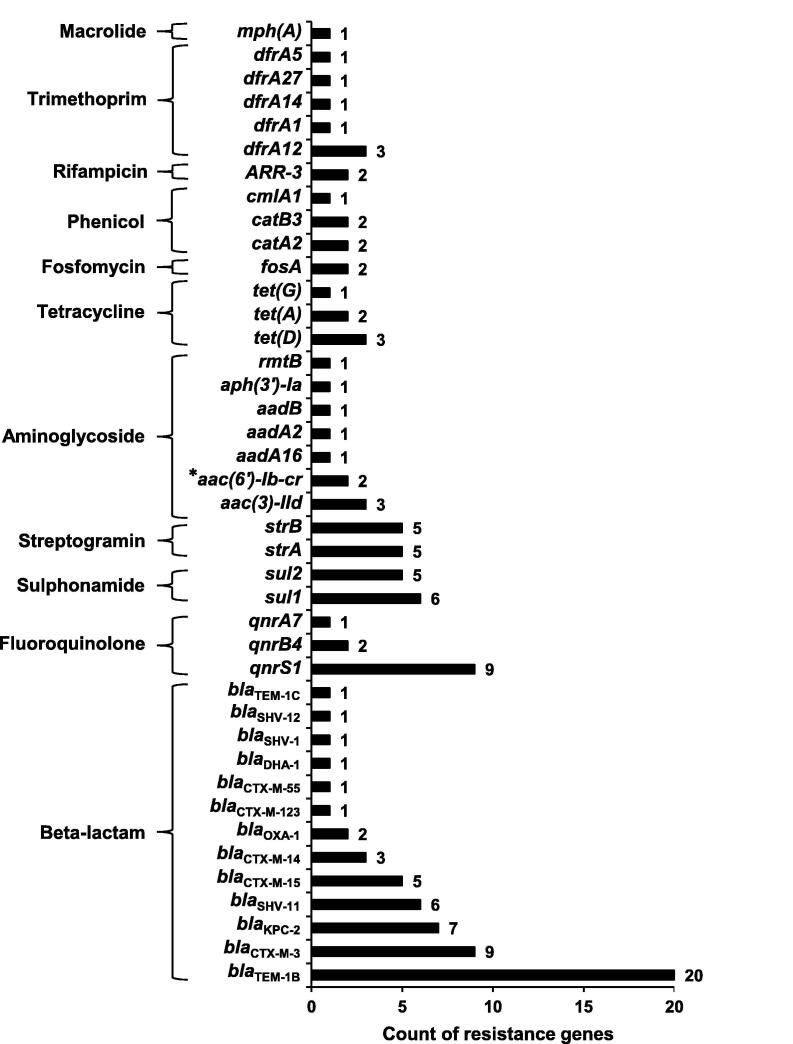
**ARGs identified in the plasmid sequence and the related antimicrobial agent classes** *aac(6′)-Ib-cr* (labeled by *) encodes an aminoglycoside acetyltransferase which can also inactivate fluoroquinolones, and thus it also belongs to fluoroquinolone resistance determinants.

We successfully revealed 124 ARGs (corresponding to 41 different ARGs), accounting for 3.6% (124/3431) of annotated ORFs in all plasmid fragments. All these ARGs could be linked to resistance against antimicrobial agents from 11 different classes, including aminoglycoside, β-lactam, fluoroquinolone, fosfomycin, macrolide, phenicol, rifampicin, sulphonamide, tetracycline, trimethoprim, and streptogramin. β-lactam resistance genes emerged more frequently than other resistance genes, in particular *bla*_TEM_ (21 times) and *bla*_CTX-M_ (19 times) (Figure 2).

Additional screening identified 317 MGEs corresponding to 61 different MGEs. These elements belong to either one of 19 IS families or class I integrons. Some of these elements are present either as a single copy or only in partial form. In contrast, numerous copies of IS*26*, Tn*3*, IS*903B*, IS*Ecp1*, and IS*Kpn19* were found, suggesting that they are transferable and most likely remain active in different genetic backgrounds. The most frequently identified IS families included the IS*6* family (73 times), Tn*3* family (47 times), IS*5* family (36 times), IS*1380* family (32 times), IS*1* family (28 times), IS*Kra4* family (23 times), and IS*3* family (21 times). Class I integrons appeared 9 times (Table 2).

**Table 2:** Families and members of ISs and integrons detected in the plasmid sequencing data

*Note*: Counts in parenthesis indicate the times of the presence of respective family and member of ISs and integrons detected in the plasmid sequencing data.

### Relationship between MGEs and ARGs

The locations of MGEs and ARGs in 82 plasmid unitigs were determined, and the interval distance between each MGE and/or ARG was also calculated, which allowed us to depict the relationship between these MGEs and/or ARGs. Hence, a relationship is assigned when the interval distance between two MGEs/ARGs or between an MGE and an ARG is < 1 kb. This cut-off value was selected because most of the well-known relationships between previously reported MGEs and ARGs occur within 1 kb, such as Tn*3*–*bla*_TEM_ and IS*Ecp1*–*bla*_CTX-M_ [11], [14], [15]. The interval distribution is shown in Figure S3.

According to the relationship analysis of our plasmid sequencing data, 90.3% (112/124) of the identified ARGs (including consecutive ARGs) are found close to MGEs, including ISs, Tns, and integrons. This result was obtained by checking the 1-kb flanking sequence of these ARGs. Subsequently, we conducted a correlation analysis for all tested ARGs to evaluate whether such a relationship is a significant correlation. To this end, we used the actual location of each ARG and its neighboring MGE on a plasmid, including the ARG–MGE pairs with an interval of > 1 kb. Pearson’s correlation coefficient (*r*) value of 0.9992 (*P* < 0.0001) confirmed a strong correlation between the tested ARGs and their neighboring MGEs, suggesting that an MGE is always located near an ARG.

### Network construction for relationships between MGEs and ARGs

**Figure 3:**
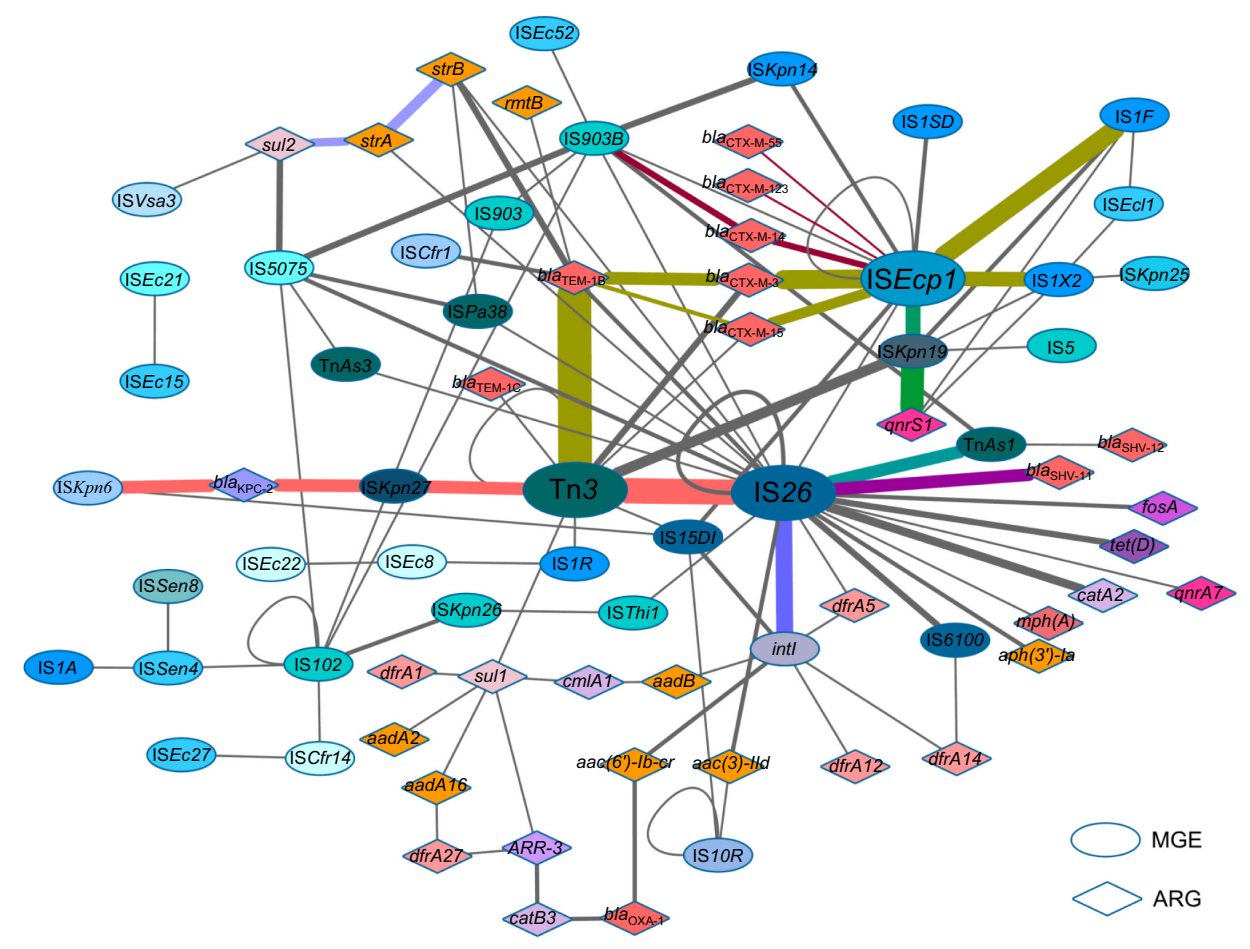
**Network of MGEs and ARGs for their relationships** The oval-shaped nodes represent MGEs; the diamond-shaped nodes represent ARGs. Nodes belonging to the same IS family or resistant to the same class of antimicrobial agents are presented in the same color. The more frequent the gene relationships are, the thicker the line is. The colored lines represent MGE or ARG clusters in which the relationships occur more frequently (more than twice per pair). The network was constructed by using Cytoscape v. 3.2.1. IS, insertion sequence; Tn, transposon.

A relationship network was constructed to illustrate MGEs or ARGs that possessed at least one relationship as a node in a network map. To do this, 39 different MGEs and 36 different ARGs were included; their relationships are represented as lines, and frequency is represented by the line thickness (Figure 3). In the network, prediction of the most important MGEs and their related ARGs was possible. For example, IS*26* had up to 64 relationships with other MGEs and ARGs, incl uding 10 MGEs and 12 ARGs. Tn*3*, IS*903B*, IS*Ecp1*, and IS*Kpn19* were also active and important in mediating HGT.

MGE–ARG relationships were further examined after establishment of the network. Those relationships that occur more frequently (more than twice per pair) are defined as “clusters”. In Figure 3, nodes connected with lines in the same colors belong to the same cluster, which facilitates tracing the dominant combinations or groups of the MGE–ARG relationships. The counts of clusters with more clinical significances in the network are listed in Table 3. *bla*_KPC-2_, the most prevalent carbapenemase gene reported in China [16], was found 6 times in the IS*Kpn6*–*bla*_KPC-2_–IS*Kpn27*–Tn*3*–IS*26* cluster. The β-lactamase gene *bla*_TEM-1_ (including TEM-1B and TEM-1C types) was present 21 times in our plasmid sequencing data and was linked to the Tn*3* transposon 17 of these 21 times. IS*Ecp1* is likely an active mobile element linking to several ARGs and MGEs. In our plasmid sequence analysis, the transfer of all CTX-M type β-lactamase genes was mediated by IS*Ecp1*. Notably, one CTX-M subtype group-9 β-lactamase gene (*i.e.*, CTX-M-14) was found in the cluster IS*Ecp1*–*bla*_CTX-M-14_-IS*903B*; in contrast, all CTX-M subtype group-1 β-lactamase genes (*e.g.*, CTX-M-3, -15, -55, and -123) were associated with IS*Ecp1* only, suggesting different transfer pathways. Moreover, the plasmid-mediated quinolone resistance determinant *qnrS1* was constantly found in the IS*Ecp1*–IS*Kpn19*–*qnrS1* or IS*Kpn19*–*qnrS1* clusters. Interestingly, some ISs were often found to be located close to other MGEs, such as IS*Ecp1* and IS*1*, IS*26* and Tn*3*, and IS*26* and int*I1*. Moreover, some ARGs could potentially link with other ARGs to form clusters, such as *sul2*–*strA*–*strB*.

**Table 3:** Count of clusters presented in the network

*Note*: Cluster means the MGE and/or ARG relationships that occur more frequently (more than twice per pair).

### IS***26***-mediated composite transposition

IS*26*, a common member of the IS*6* family with a full length of 820 bp containing two 14-bp TIRs, was found to be the predominant MGE in our plasmid sequencing data. IS*15DI* and IS*6100*, the other two IS*6* family members with similar sequences and functions to IS*26*, were also identified. All of them have been reported to generate 8-bp TSDs when a transposition event occurs. We examined all 8-bp flanking sequences of the 62 full copies of IS*26*, IS*15DI*, and IS*6100* in 27 unitigs. 44 of 123 8-bp sequences were found to possess identical patterns, suggesting that replicative transposition events had occurred between these unitigs, likely within or between plasmids (Figure S4).

**Figure 4:**
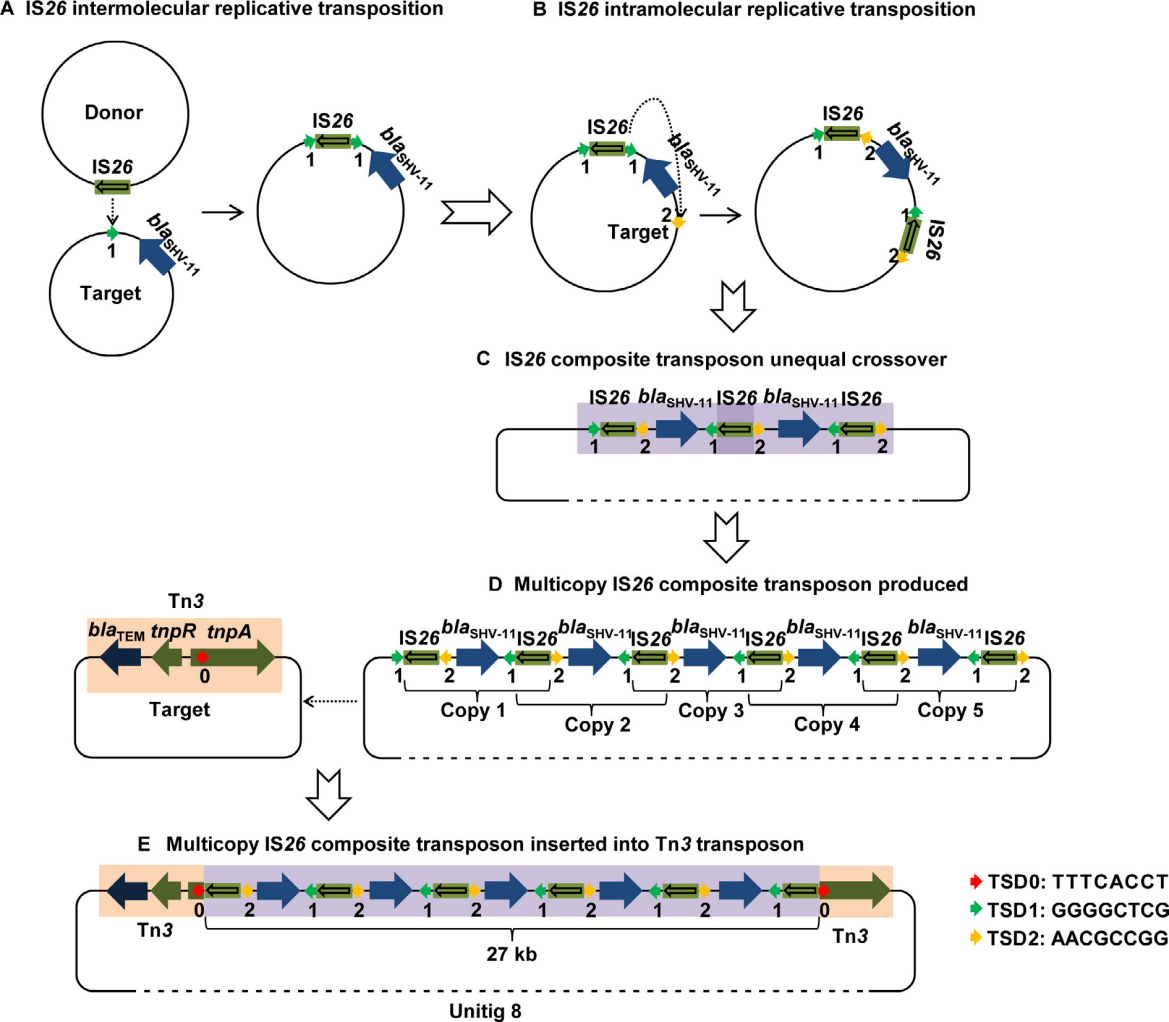
**The entire process of multicopy IS*26* composite transposon generation deduced based on tracing the 8-bp TSD distribution** **A.** First, an IS*26* intermolecular replicative transposition event occurs, whereby IS*26* from the donor plasmid attacks the target site “1” in another plasmid near *bla*_SHV-11_, resulting in duplication of IS*26* and the 8-bp TSD1 (GGGGCTCG). **B.** Second, IS*26* with TSD1 launches another intramolecular attack and copies itself to the other side of *bla*_SHV-11_, leading to the duplication of the 8-bp (TSD2, AACGCCGG) at the target site “2”. **C.** The unequal crossover combination occurs, producing a copy of the IS*26* composite transposon. **D.** The formation of the IS*26* composite transposon with multiple copies in a row. **E.** Finally, the multicopy IS*26* composite transposon attacks the target site “0” within the transposase (*tnpA*) gene of the Tn*3* transposon and inserts the 27-kb fragment along with the duplication of the 8-bp TSD0 (TTTCACCT). TSD, target site duplication.

A typical hallmark of multicopy IS*26* was noted for a composite transposition event in unitig 8, one of the largest fragments among our plasmid sequences. We found an approximately 27-kb multicopy IS*26* composite transposon containing six IS*26* copies and five *bla*_SHV-11_ genes arranged in an interlaced order as well as two partial Tn*3* fragments. We attempted to deduce the entire generation process of the IS*26*-mediated composite transposon (Figure 4). First, an IS*26* intermolecular replicative transposition event occurred, whereby an IS*26* element from the donor plasmid attacked the target site “1″ in another plasmid near *bla*_SHV-11_, resulting in the duplication of IS*26* and the 8-bp TSD1 (GGGGCTCG, Figure 4A). Second, IS*26* with TSD1 launched another intramolecular attack and copied itself into the other side of *bla*_SHV-11_, leading to the duplication of the 8-bp TSD2 (AACGCCGG) at the target site “2” (Figure 4B). Formation of the IS*26* composite transposon produced one to multiple copies in a row, followed by an unequal crossover combination (Figure 4C and D). Finally, the multicopy IS*26* composite transposon attacked the target site “0” within the *tnpA* gene (encoding a transposase) of the Tn*3* transposon and inserted the 27-kb fragment within *tnpA*, along with the duplication of the 8-bp TSD0 (TTTCACCT) (Figure 4D and E). These TSDs provided adequate evidence of the complicated transposition process and IS*26*-mediated evolution within or between the plasmids.

## Discussion

Plasmids are considered as not only the key vector for genetic exchange but also an important contributor to the novelty and evolution of prokaryotic genomes [17]. Using clinically isolated MDR *K*. *pneumoniae* strains, this study characterized the horizontal transfer profile of MGEs and ARGs in conjugative plasmids using long-read high-throughput sequencing. A relationship map was constructed for MGEs and ARGs to describe the manner of resistance dissemination and to track a composite transposition event.

The 53 *K*. *pneumoniae* clinical isolates selected in the current study presented MDR phenotypes and clonal diversity, harbored large and diverse plasmids, and were able to transfer their plasmids along with antimicrobial resistance profiles to a recipient via conjugation, guaranteeing the abundant plasmid sequence data for genetic analysis. Direct DNA extraction using mixed broth culture facilitated the acquisition of plasmid DNA in just one step followed by the sequencing procedure, which overcomes the high complexity of individual complete sequencing.

We noticed that 124 ARGs and 317 MGEs occupied a relatively large portion of all annotated ORFs (441/3431, 12.9%) in the plasmid sequencing data. Most ARGs that present resistance against 11 classes of antimicrobial agents were mediated by MGEs, exhibiting the high transferability of plasmid-mediated resistance genes. Moreover, some MGEs, such as IS*26*, Tn*3*, IS*903B*, IS*Ecp1*, and IS*Kpn19*, appeared frequently in our tested plasmid pool, demonstrating that these MGEs are easily transferred or can jump to other sites among plasmids or even between plasmid and chromosome, in most cases, along with ARGs.

Due to the complexity of plasmid annotation, in our MGE and ARG relationship analysis, rather than solely taking two consecutive genes to constitute a relationship, we used a 1-kb interval cut-off between each MGE and/or ARG to define a relationship that benefited our further analysis. In this way, we constructed a network map to explore the relationships between MGEs and/or ARGs in our plasmid sequencing data. This newly developed method will significantly advance our understanding of the spread and emergence of antimicrobial resistance.

It has been known for over 40 years that ARGs are often associated with MGEs [18]. This has been recently reinforced by genome sequencing of clinical isolates [11]. For instance, *bla*_CTX-M_, a gene encoding an extended-spectrum β-lactamase, is often located downstream of IS*Ecp1* [14], [15]. Among carbapenemase genes, *bla*_KPC_ is typically carried by the Tn*3* family transposon Tn*4401* [19], [20]. As expected, our plasmid sequencing results suggest that the plasmids present within the clinical environment harbor a diverse array of MGEs and ARGs. More than 10% of the genes in the plasmid sequences were transfer- or resistance-related genes. Some MGEs have been found to play an important role in the dissemination of ARGs. For example, IS*26* is easily inserted into other plasmid locations, making it possible to exchange large fragments between two plasmids or between a plasmid and a chromosome via multicopy IS*26* element recombination [21]. In the current study, IS*26* was found to be associated with many other MGEs, such as Tn*3* and int*I1*, and with many different ARGs, such as *bla*_SHV_, *fosA*, *aph(3′)-Ia*, and *catA2*. The Tn*3* transposon generally consists of a Tn*3* transposase, a resolvase, and a TEM β-lactamase, which mediates β-lactam antibiotic resistance dissemination. In China, the carbapenemase gene *bla*_KPC-2_ is usually located within a Tn*3*–Tn*4401* composite transposon in a consecutive gene order IS*Kpn6*–*bla*_KPC-2_–IS*Kpn27*–Tn*3*–IS*26* [22]. Similarly, the β-lactamase gene *bla*_CTX-M_ is transferred by IS*Ecp1* or IS*903B* [14], [15]. We also noticed that two large subtypes (groups 1 and 9) of CTX-M types utilize different transposition pathways. CTX-M subtype group-1 (CTX-M-3, -15, -55 as prevalent members) is solely mediated by IS*Ecp1*, whereas CTX-M subtype group-9 (*e.g.,* CTX-M-14) is often transferred by IS*Ecp1* and IS*903* together. By constructing the MGE–ARG network, this study is the first to report some new MGE–ARG relationships, such as IS*Kpn19*–*qnrS1*, IS*Ecp1*–IS*1*, IS*26*–Tn*3*, IS*26*–int*I1*, *sul2*–*strA*–*strB*, and IS*26*–*bla*_SHV_. Among these new relationships, the most prevalent plasmid-mediated quinolone resistance determinant *qnrS1* should be given more attention with regard to its rapid transposition mediated by the active IS*Kpn19* element.

Many different ARGs were found in compound transposons bound by IS*26* on a plasmid, and multiple and extensively resistant Gram-negative bacteria often simultaneously carry several ARGs and multiple copies of IS*26*. The transposition mechanism of IS*26* is generally regarded to involve replicative transposition and cointegrate formation. Indeed, the high copy number observed in this study reflects the high activity of IS*26*, which is consistent with previous reports on other clinical Enterobacteriaceae isolates [23], [24]. In this study, 8-bp TSDs flanking IS*26* were used to investigate the transposition pathway of mobile elements. TSDs are genomic signatures of transposition events mediated by DDE transposases, and their length is a characteristic property of the transposon. The entire likely process of IS*26*-mediated generation of multicopy composite transposons was deduced to illustrate how to track the evolution of resistance plasmids based on TSD patterns.

A major limitation of our study, like many others, is the lack of systemic evaluation and validation of this new analytic methodology for data processing. The findings reported in this study are based on a novel pooled long-read sequencing method. The presence of plasmids with similar structures might have induced assembly artefacts and consequently obscured the results. To address this issue, we have employed diverse and deliberate sequence analysis and hope to draw a rough network map of MGEs and ARGs, which can facilitate a further understanding of the novel HGT aspect in a clinical environment.

## Materials and methods

### Bacterial isolates

*K*. *pneumoniae* MDR strains were isolated from 38 wards of 21 departments in a tertiary referral hospital (The First Affiliated Hospital, Zhejiang University School of Medicine) in Hangzhou, China, over a 9-month period in 2009. Identification of bacterial species was carried out using a Vitek II system (BioMerieux, Marcy, France), and 53 of 84 collected isolates were able to transfer their plasmids to transconjugants and used for further analysis.

### Plasmid conjugation

Plasmid conjugation was carried out via filter mating assays. Rifampin-resistant *Escherichia coli* EC600 was used as the recipient strain. Exponential-phase LB broth cultures of the donor and recipient strains were mixed at a volumetric ratio of 1:1. A 20-μl aliquot of this mixture was then transferred to the surface of a 0.22-μm GSWP-type nitrocellulose membrane (Millipore, Tullagreen, Ireland) and incubated at 35 °C for 18 h. Transconjugants harboring the donor plasmids were selected on Mueller-Hinton agar plates (MH, Oxoid, Basingstoke, UK) supplemented with ampicillin (50 μg/ml) and rifampin (700 μg/ml).

### PFGE typing

After digestion with *Xba*I (Takara, Kusatsu, Japan), a PFGE assay was carried out on the clinical isolates using the contour-clamped homogenous electric field (CHEF; Bio-Rad, Hercules, CA) technique [25]. Briefly, bacterial DNA was separated by electrophoresis through 1% agarose III (Sangon, Shanghai, China) using 0.5× Tris-borate-EDTA buffer for 22 h. The electrophoresis was conducted at 14 °C, 6 V/cm with alternating pulses at a 120° angle and in a 5–35-s pulse time gradient. *Salmonella enterica* serotype Braenderup H9812 was used as a control and size marker [26].

### S1 nuclease digestion assay

An S1 nuclease digestion assay was performed on the transconjugants to estimate the size of the plasmids in the absence of genomic DNA using PFGE. Agarose gel plugs containing bacterial cells were incubated for 50 min with 20 U of S1 nuclease (Takara, Shiga, Japan). The digested plugs were then processed by PFGE using the CHEF apparatus with a 2.16–63.8-s pulse time gradient for 20 h.

### Antimicrobial susceptibility testing

The antimicrobial susceptibility profiles of the original clinical isolates and their *E*. *coli* transconjugants were determined using the K-B agar diffusion method, following the Clinical and Laboratory Standards Institute (CLSI) guidelines [27]. The antimicrobial agents tested in the assay included ampicillin, ampicillin/sulbactam, piperacillin, piperacillin/tazobactam, cefazolin, cefuroxime, ceftazidime, cefotaxime, cefepime, cefoxitin, amoxicillin/clavulanic acid, cefoperazone/sulbactam, meropenem, imipenem, aztreonam, ciprofloxacin, gentamicin, amikacin, trimethoprim-sulphamethoxazole, and tetracycline. *E*. *coli* ATCC25922 was used as the quality control.

### Pooled plasmid sequencing

Each transconjugant was cultured overnight in LB broth and adjusted to OD = 1.0 at a wavelength of 600 nm. Then, 5 ml of broth was collected from each isolate and mixed up before commencing plasmid DNA extraction using a Plasmid Midi Kit (Qiagen, Hilden, Germany). A pool of conjugative plasmid DNA acquired from an *E*. *coli* transconjugant mixture was sequenced via the SMRT technique using the PacBio RS II platform (Pacific Biosciences, Menlo Park, CA) and assembled with its affiliated assembly tool. The assembled fragments designated as unitigs were annotated using the prokaryotic gene prediction tool Prokka [28] and BLAST (https://www.ncbi.nlm.nig.gov/blast). The complete genome of *E*. *coli* strain DH1 was employed to filter assembled unitigs of transconjugant chromosome sequences. The remaining unitigs were deemed conjugative plasmid fragments and double-checked based on their gene annotation. Some plasmid fragments with flanking repetitive sequences were cyclized, and redundant sequences were deleted. The plasmid unitigs were then scanned for ARGs and plasmid replicons using ResFinder 2.1 (https://cge.cbs.dtu.dk/services/ResFinder/) [29] and PlasmidFinder 1.3 (https://cge.cbs.dtu.dk/services/PlasmidFinder/) [30], respectively, on the Center for Genomic Epidemiology (CGE) server. Transposon and IS elements were scanned using the ISFinder database (http://www-is.biotoul.fr/) [31]. The integrase genes of integron type I, II, and III elements were scanned with BLAST tools.

### ARG–MGE correlation analysis

To evaluate correlations between ARGs and MGEs, we used the actual location of each ARG and its neighboring MGE on a plasmid, including the ARG**–**MGE pairs with an interval of more than 1 kb for the Pearson’s correlation coefficient (*r*). An *r* value of 0–0.09 suggested no correlation; *r* = 0.1–0.5 for low correlation; *r* = 0.5–0.8 for high correlation; and *r* = 0.9–1 for a very strong correlation. If an MGE is constantly found to be located near an ARG, they are considered correlated. GraphPad Prism 7.0 (La Jolla, CA) was utilized for the correlation analysis. A two-tailed Student’s *t*-test was employed to calculate the significance of the correlation analysis above, where *P* < 0.001 indicates strong evidence to reject the null hypothesis (*r* = 0).

## Data availability

The plasmid assembly unitigs generated by the PacBio platform in this study were deposited in the European Nucleotide Archive (ENA: PRJEB23499) that can be accessed at https://www.ebi.ac.uk/ena/, and also in the Genome Sequence Archive [32] at the National Genomics Data Center, Beijing Institute of Genomics, Chinese Academy of Sciences / China National Center for Bioinformation (GSA: CRA003487) that are publicly accessible at https://bigd.big.ac.cn/gsa/.

## CRediT author statement

**Yan Jiang:** Conceptualization, Formal analysis, Data curation, Writing - original draft. **Yanfei Wang:** Investigation. **Xiaoting Hua:** Visualization, Data curation. **Yue Qu:** Visualization, Writing - review & editing. **Anton Y. Peleg:** Writing - review & editing. **Yunsong Yu:** Conceptualization, Supervision. All authors read and approved the final manuscript.

## Competing interests

The authors have declared no competing interests.

## Supplementary material

Supplementary data 1 **Characteristics of conjugative plasmids of all *K. pneumoniae* transconjugants. A.** The plasmid bands of all transconjugants after S1 nuclease digestion and PFGE. M, marker (*Salmonella enterica* serotype Braenderup H9812). PFGE, pulsed-field gel electrophoresis. **B.** Distribution of the lengths of 53 conjugative plasmids. The lengths were estimated based on the plasmid bands shown in A.

Supplementary data 2 **Proportion of resistant, intermediate, and susceptible isolates among the 53 clinical *K. pneumoniae* isolates**.

Supplementary data 3 **Interval distribution of known MGE–ARG relationships.** The *X*-axis shows the interval distance (bp) between MGEs and/or ARGs that are well known from the previous literature, such as IS*Kpn6*–*bla*KPC-2–IS*Kpn27*–Tn*3*, Tn*3*–*bla*TEM, and IS*Ecp1–bla*CTX-M–IS*903B* [11,14,15,22]. The *Y*-axis shows the count of these relationships detected in our plasmid sequencing data.

Supplementary data 4 **The 8-bp flanking sequences of IS*26*, IS*15DI*, and IS*6100* in 27 unitigs showing the potential TSD sites.** For each unitig, a pair of short segment represents the 8-bp sequences just flanking the IS*26*, IS*15DI*, or IS*6100* (the black, dark red and yellow solid arrows, respectively). The 8-bp sequences that are found more than once in all 27 unitigs are shown in color, and the same sequences shown in the same color represent the sites where potential TSD occur. In unitig 16, the second 3′ 8-bp sequence flanking IS*6100* is missing, which is indicated with a blank box.
