# Supplementary figures and images for: Pooled Plasmid Sequencing Reveals the Relationship Between Mobile Genetic Elements and Antimicrobial Resistance Genes in Clinically Isolated Klebsiella pneumoniae
Source: Genomics Proteomics Bioinformatics. 2020 Dec 30;18(5):539–48. doi: 10.1016/j.gpb.2020.12.002 (PMC8377239; doi:10.1016/j.gpb.2020.12.002)

**A**

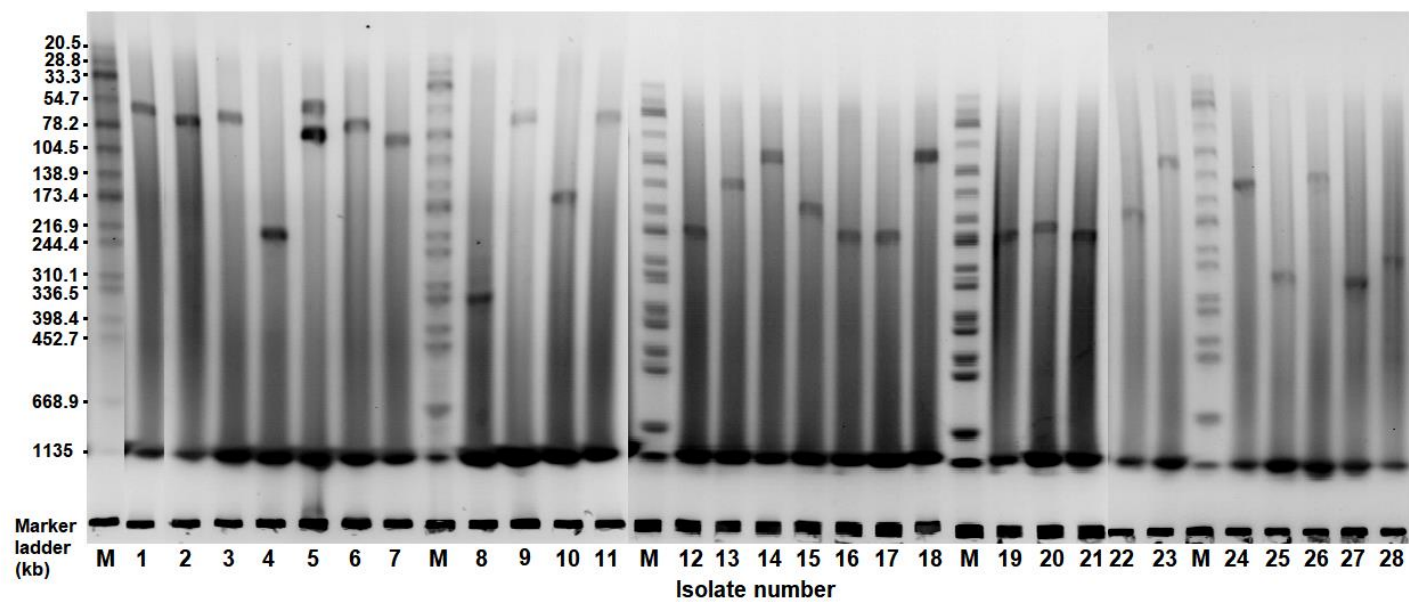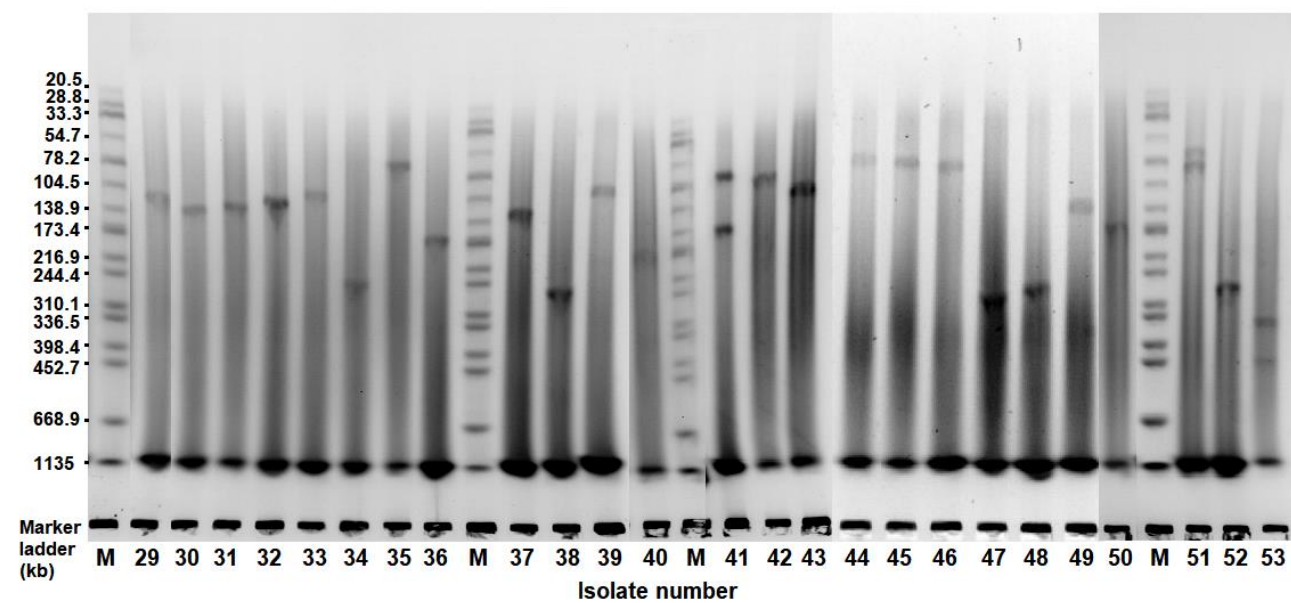

**B**

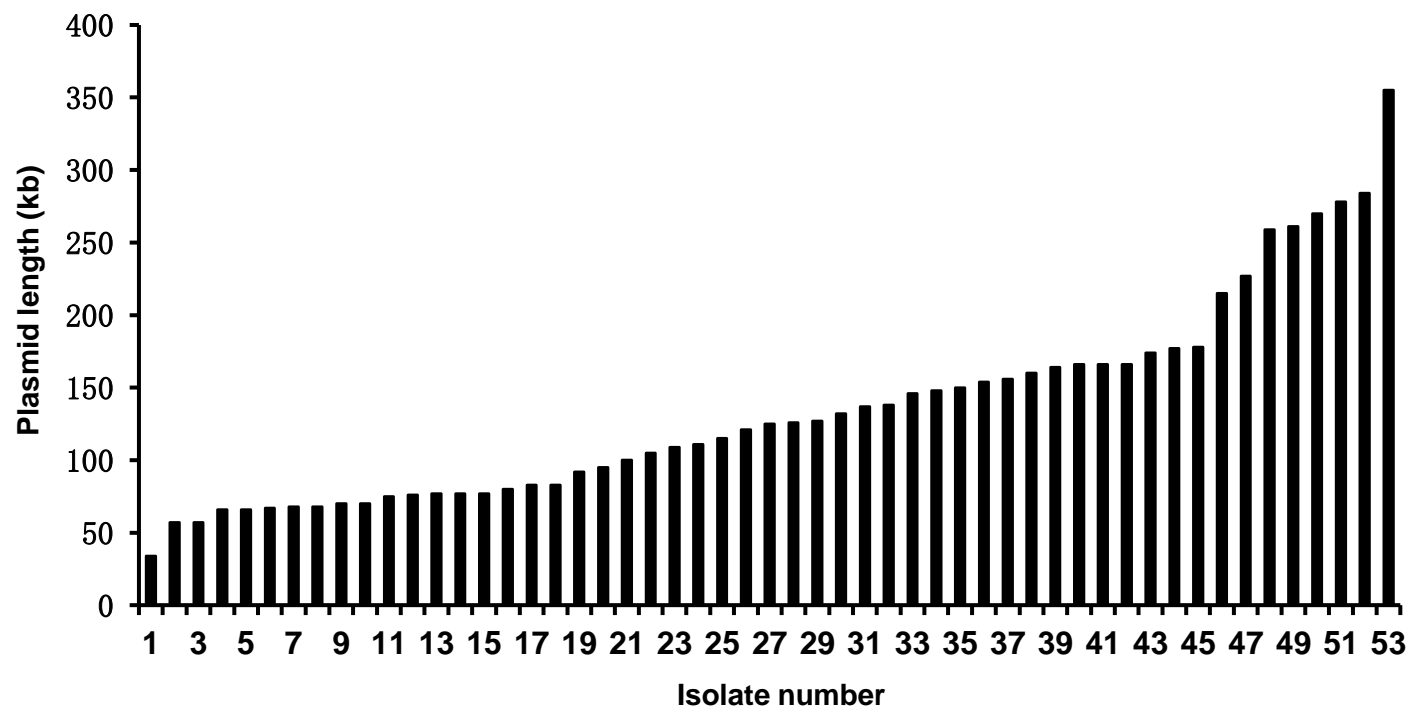

Supplement: Supplementary data 1 — Characteristics of conjugative plasmids of all K. pneumoniae transconjugants. A. The plasmid bands of all transconjugants after S1 nuclease digestion and PFGE. M, marker (Salmonella enterica serotype Braenderup H9812). PFGE, pulsed-field gel electrophoresis. B. Distribution of the lengths of 53 conjugative plasmids. The lengths were estimated based on the plasmid bands shown in A. [file mmc1.pdf]

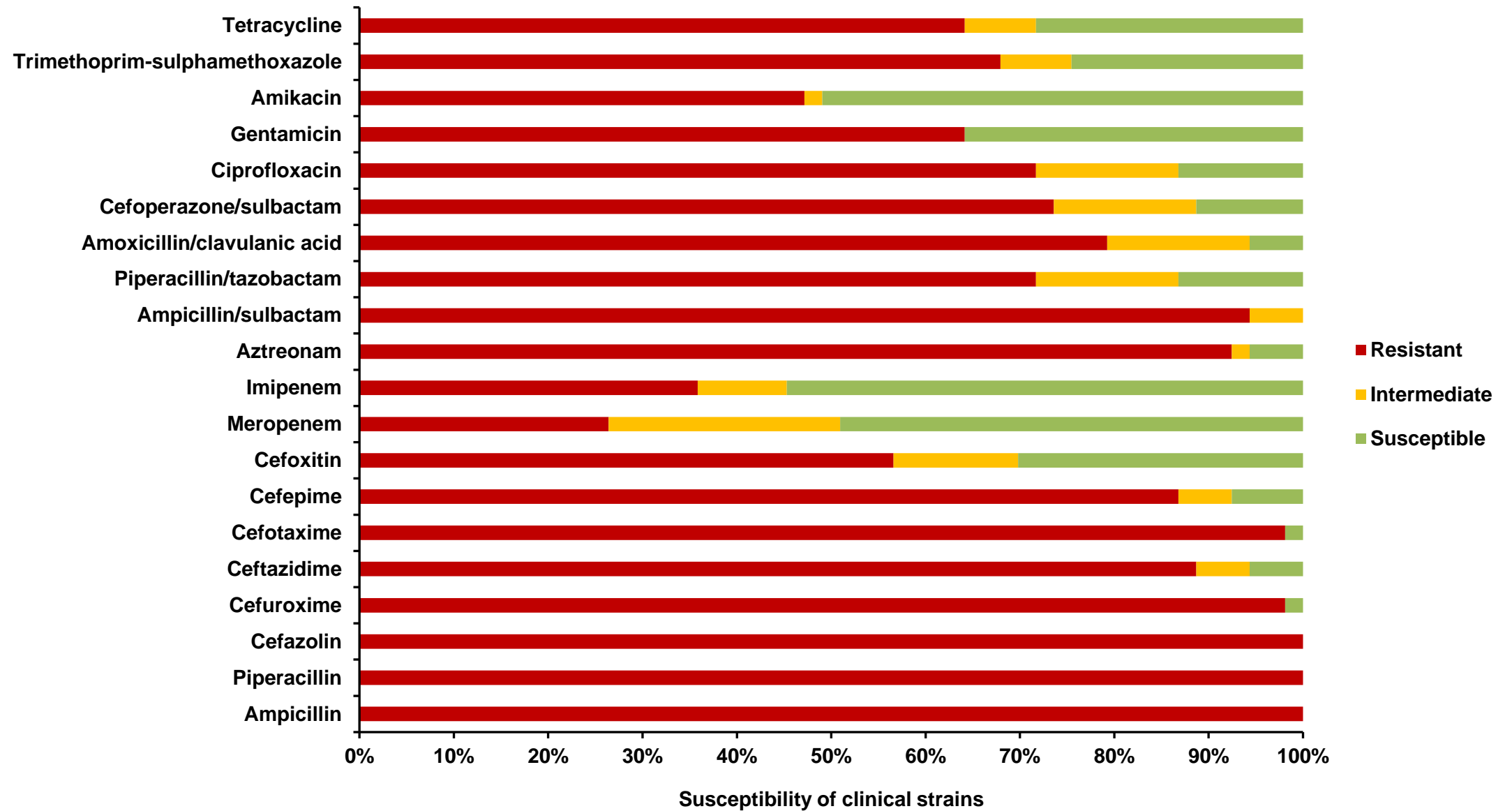

Supplement: Supplementary data 2 — Proportion of resistant, intermediate, and susceptible isolates among the 53 clinical K. pneumoniae isolates. [file mmc2.pdf]

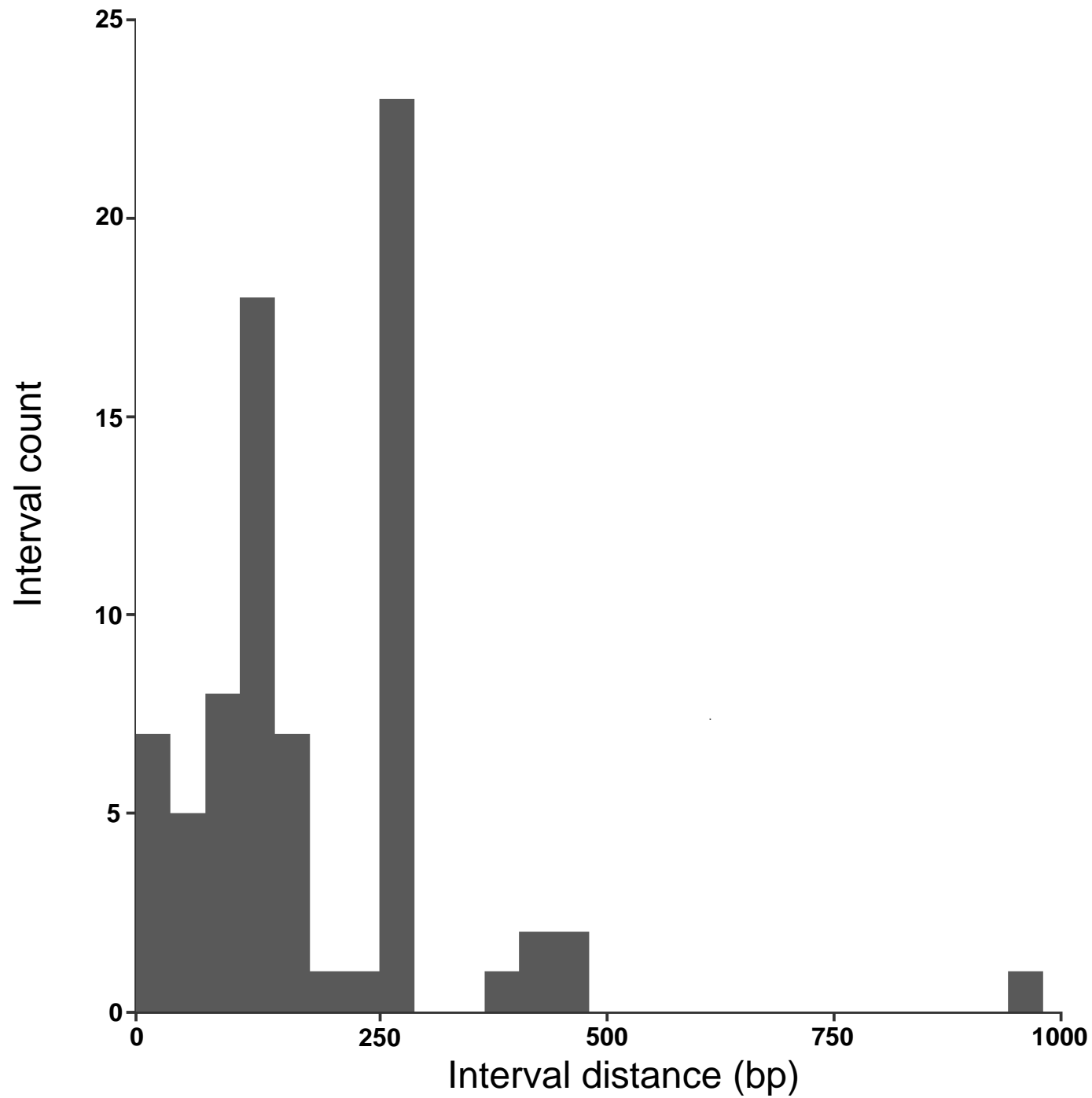

Supplement: Supplementary data 3 — Interval distribution of known MGE–ARG relationships. The X-axis shows the interval distance (bp) between MGEs and/or ARGs that are well known from the previous literature, such as ISKpn6–blaKPC-2–ISKpn27–Tn3, Tn3–blaTEM, and ISEcp1–blaCTX-M–IS903B [11,14,15,22]. The Y-axis shows the count of these relationships detected in our plasmid sequencing data. [file mmc3.pdf]

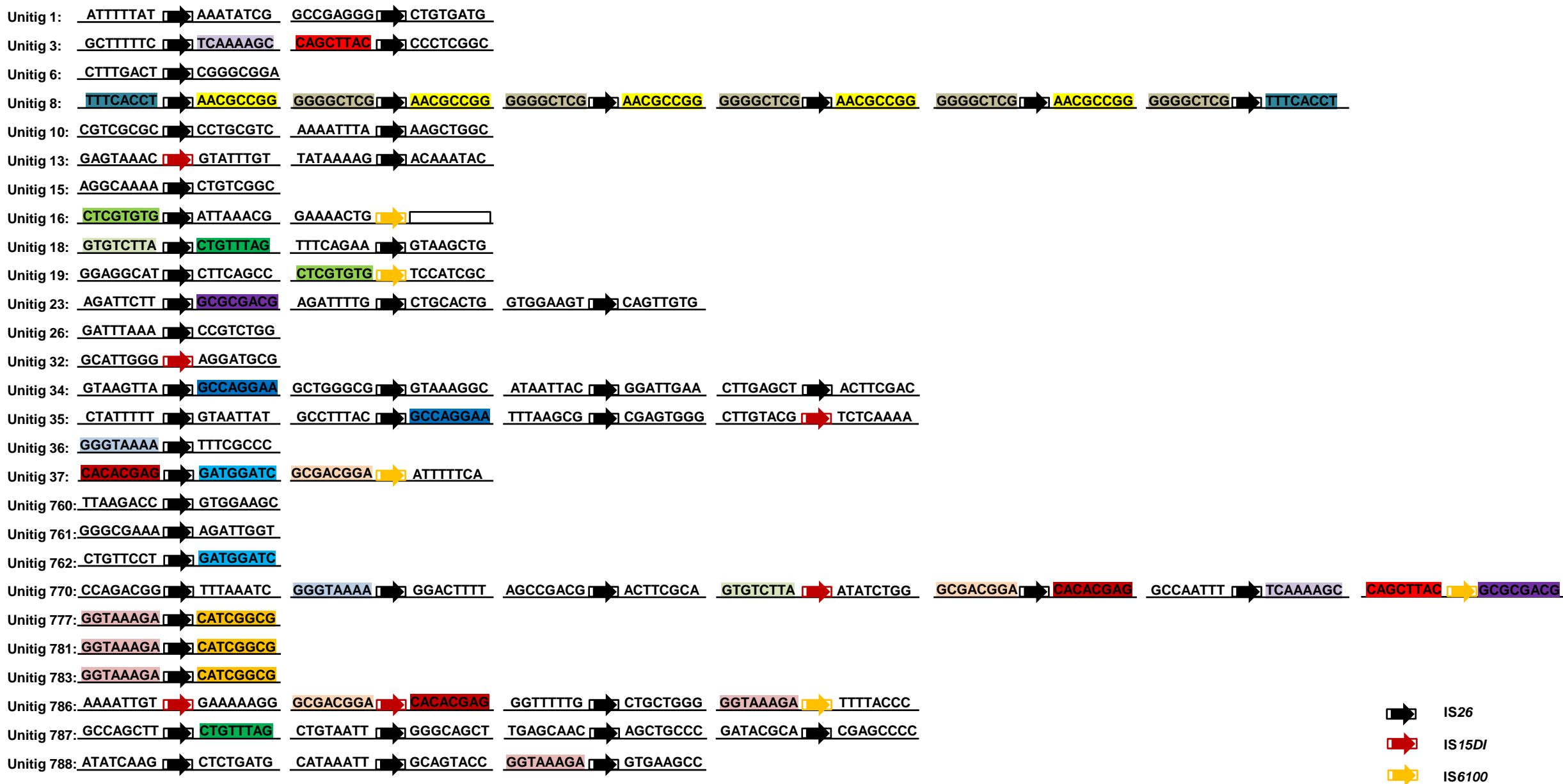

Supplement: Supplementary data 4 — The 8-bp flanking sequences of IS26, IS15DI, and IS6100 in 27 unitigs showing the potential TSD sites. For each unitig, a pair of short segment represents the 8-bp sequences just flanking the IS26, IS15DI, or IS6100 (the black, dark red and yellow solid arrows, respectively). The 8-bp sequences that are found more than once in all 27 unitigs are shown in color, and the same sequences shown in the same color represent the sites where potential TSD occur. In unitig 16, the second 3′ 8-bp sequence flanking IS6100 is missing, which is indicated with a blank box. [file mmc4.pdf]
